# Cyclophosphamide "metronomic" chemotherapy for palliative treatment of a young patient with advanced epithelial ovarian cancer

**DOI:** 10.1186/1471-2407-7-65

**Published:** 2007-04-15

**Authors:** Riccardo Samaritani, Giacomo Corrado, Enrico Vizza, Carlo Sbiroli

**Affiliations:** 1 Division of Medical Oncology, San Carlo IDI Sanità Hospital, Rome, Italy; 2 Division of Gynaecologic Oncology, National Cancer Institute "Regina Elena", Rome, Italy

## Abstract

**Background:**

Evaluation of the clinical efficacy and tolerance of metronomic chemotherapy as salvage therapy in a young patient with advanced, platinum resistant, ovarian carcinoma and bad performance status.

**Case presentation:**

We tried palliative chemotherapy with daily low dose oral cyclophosphamide with a patient suffering from stage IIIC ovarian cancer that responded to daily cyclophosphamide (CTX) after no response to chemotherapy with paclitaxel and carboplatin as first line and progression after second line with topotecan.

The progression-free survival time on daily low dose oral cyclophosphamide treatment was 65 months without side effects. She was well during the chemotherapy and lived a normal working and social life.

**Conclusion:**

We think that use of low dose of oral CTX should be investigated further as a strategy against tumour progression after standard chemotherapy in patients who are platinum resistant with poor performance status.

## Background

Ovarian cancer remains the most common cause of death from a gynaecologic malignancy. In 2005, it is estimated that over 22,000 women will develop ovarian cancer and 16,210 will die as a result [1].

Current treatment of ovarian cancer entails a combination of surgery and chemotherapy. Currently, 1st-line chemotherapy consists of a combination of carboplatin and paclitaxel to which approximately 80% of women respond [2].

However, despite aggressive surgery and chemotherapy, more than 80% of patients will relapse and will then be treated with second line chemotherapy with objective responses in about 20% of patients and even lower percentages of complete responses. This is why the main goal of second and third line chemotherapy is palliative care with the aim to prolong time to progression and to improve quality of life.

Several chemotherapeutic regimens have been used as single agents in phase II trials with patients previously treated with cisplatin including paclitaxel, topotecan, liposomal doxorubicin, gemcitabine and oxaliplatin, showing objective response rates ranging from 10–50% and different toxicity profiles [3].

Many factors play a role when choosing a drug, such as patient compliance, toxicity of previous treatments and treatment costs. Because, at present there are no parameters to predict response to second or third line chemotherapy, the need for less toxic and more efficacious outpatient chemotherapeutic regimens as salvage therapy for advanced ovarian carcinoma is significant.

Recent experimental studies have shown that certain cytotoxic agents, when administered at low and frequent doses are more effective in targeting tumour endothelium than large single bolus doses followed by long rest periods. This is because intra-tumoural vascular endothelial cells, in contrast to the endothelium of quiescent mature blood vessels of normal adult tissues, proliferate rapidly and are vulnerable to cytotoxic agents. However, the long interval between cycles of conventional chemotherapy permits the survival and re-growth of a small number of endothelial cells, allowing tumour angiogenesis to persist. Continuous, low-dose chemotherapy, on the other hand, enhances the anti-angiogenic effect of some cytotoxic agents and may also enhance pro-apoptotic effect in both dividing tumour cells and endothelial cells. A further advantage in the use of continuous low-dose chemotherapy is that it minimises the toxic effects of drugs, allowing more combinations of potentially synergistic selective inhibitors of angiogenesis. The use of dosing regimens that involve the frequent administration of small doses of drugs without extended rest periods has been termed "metronomic" or "high time" chemotherapy [4]. For these reasons we evaluated the clinical efficacy and tolerance of metronomic chemotherapy as salvage therapy in a young patient with advanced ovarian carcinoma platinum resistance and bad performance status.

## Case presentation

**Figure 1:**
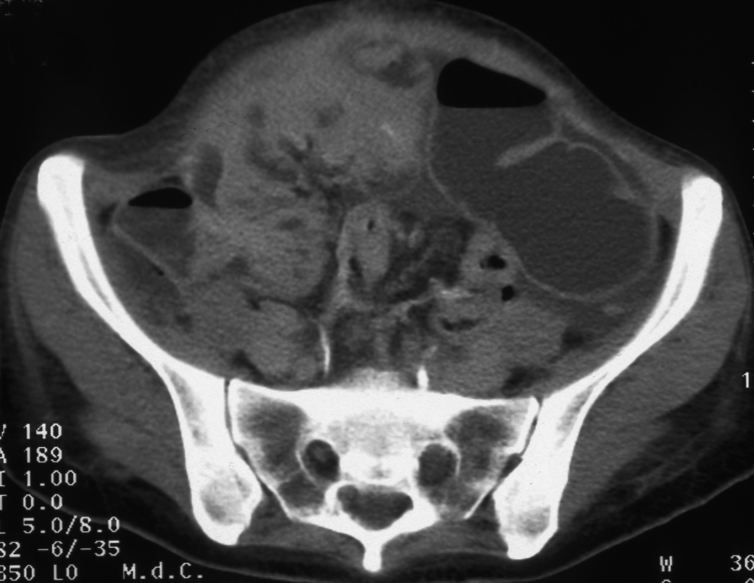
Computer tomography (CT) scan of advanced ovarian cancer before treatment showing conglomeration of the bowel.

In January 2001 a 36 year old woman, nulliparous, menarche at 13 years with a regular cycle came to our Institution because of abdominal pain and increase of abdominal volume. Serum tumour marker tests revealed abnormally high levels of CA-125 (182 IU/ml), but normal levels of CA-19-9 (20,68 IU/ml), CA 15-3 (16 IU/ml) and CEA (1.1 ng/ml). Ultrasound scan (US) and Computer tomography (CT) of the pelvis was done at our surgical department and revealed a moderate amount of ascites and a conglomeration of the bowel [Fig 1].

Explorative laparotomy revealed one litre of ascites, diffuse peritoneal carcinomatosis and a solid tumour originating from the ovary that formed a mass with extensive adhesions to the uterus, sigmoid colon, and rectum in the pouch of Douglas. There were also numerous metastatic tumours with a diameter of about 3 cm in the greater omentum and the retroperitoneum. Based on these findings, we decided to only perform biopsy of the tumour. Pathologic examination revealed poorly differentiated serous adenocarcinoma arising from the ovary, FIGO stage IIIC

After surgery, six courses of chemotherapy with paclitaxel (175 mg/m^2^) and carboplatin (CBDCA: AUC 5) were given every 3 weeks.

At the end of chemotherapy a re-evaluation of the situation by CT scan and serum marker CA 125 (102 IU/ml) showed no response to therapy. For this reason we started with a second line of chemotherapy with topotecan (1,5 mg/m^2^) for 5 days every 3 weeks. Unfortunately, fifteen days from the first cycle of chemotherapy the patient underwent surgery again due to an intestinal occlusion.

The second laparotomy showed that the pelvis and the abdomen were completely obstructed by a tumour mass that incorporated the bowel with widespread peritoneal carcinomatosis of diaphragmatic peritonetoneum and on liver surface. This situation with an increase of serum marker Ca 125 (300 IU/ml) showed a clear progression of the disease. Given the extension we decided to perform only a colostomy.

Even if the patient only had one cycle of second line chemotherapy, it was not possible to verify response to further treatment with topotecan, because of the general deterioration with marked weight loss and anaemia grade 2 (classification by the NCI-CTC).

After thorough discussion with the patient, informed consent was obtained and further salvage chemotherapy was started in August 2001. The regimen of a palliative chemotherapy was oral cyclophosphamide (50 mg) every day plus support therapy with epoetin alpha (10000 IU) every day plus vitamins.

Even if evaluation with CT scan and serum marker Ca 125 (90 IU/ml), 3 months after colostomy, showed stabilization of the disease, surprisingly, the patient improved with disappearance of anaemia and weight increase.

**Figure 2:**
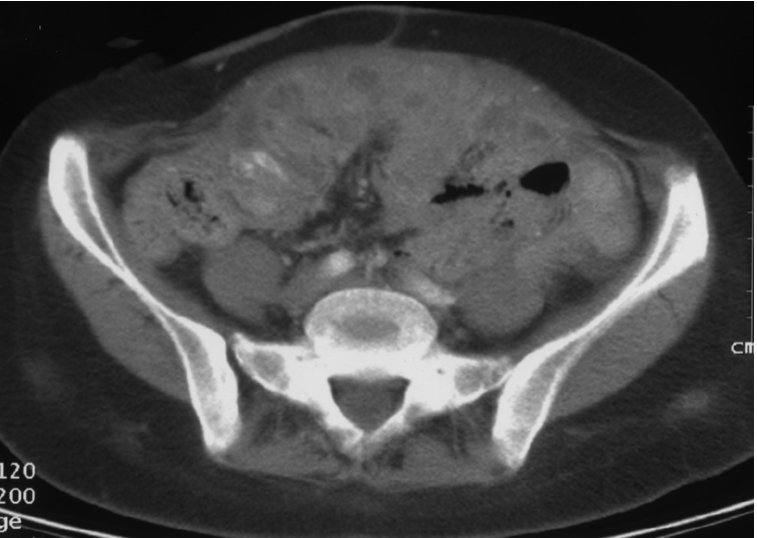
Computer tomography (CT) scan of advanced ovarian cancer after 65 months after diagnosis showing stable disease without bowel occlusion due to successful colostomy.

For 65 months after the diagnosis of ovarian cancer FIGO stage IIIC platinum resistant the patient continued the chemotherapy with oral cyclophosphamide (50 mg) every day plus vitamins without side effects. She had a stabilization of disease [Fig 2] with a normal blood exams, normal liver and kidney function tests, stabilization of serum marker Ca 125 (50 IU/ml) and a good performance status (ECOG = 0). In fact she was well and lived a normal working and social life during chemotherapy.

In July 2006 the patient came to our institution because of bladder haemorrhage. We performed a cystoscopy that showed external compression of the bladder with an internal formation of the bladder bottom which bled easily. The biopsy showed a carcinoma of ovarian origin that revealed a progression of disease.

Unfortunately, the patient died from a severe bladder haemorrhage before trying an other palliative chemotherapy line.

## Discussion

Despite advances in ovarian cancer chemotherapy regimens, there is a high rate recurrence. Second-line ovarian cancer therapy often aims at prolonging survival and time to symptom recurrence. When disease progression or recurrence occurs after primary chemotherapy, choice of subsequent therapy must be individualized.

Due to poor response rates with repeat dosing with platinum agents, second-line treatment for platinum-resistant patients usually involves a cytotoxic agent that uses a different mechanism. These agents include topoisomerase inhibitors (etoposide, topotecan), alkylating agents (ifosfamide, cyclophosphamide), and other chemotherapies (doxorubicin, gemcitabine, docetaxel).

At this time, no one drug combination has been shown to be more efficacious; thus, currently, there is no standard-of-care salvage treatment recommendation.

Moreover, these agents used at the maximum tolerated doses may be difficult to administer and are often associated with severe side-effects, sometimes requiring hospitalization.

Not surprisingly, therefore, patient attitudes to toxic chemotherapy regimens for advanced, platinum resistant, ovarian cancer are often negative, as the adverse effects of treatment often seem to outweigh any potential benefits. Thus, the introduction of newer approaches, having improved or at least equivalent efficacy but reduced toxicity, are highly desirable. Therapy must be individualized according to previous response, toxicities, and patient wishes.

Instead of only using short bursts of toxic MTD chemotherapy with long breaks to allow recovery from the harmful side effects, there is now a shift in thinking towards the view that more compressed or accelerated schedules of drug administration using much smaller individual doses than the MTD would be more effective, not only in terms of reducing certain toxicities, but perhaps even improving anti tumour effects as well [5]. The most recent refinement of this concept is called "metronomic" chemotherapy, which refers to the frequent, even daily, administration of chemotherapeutics at doses significantly below the MTD, with no prolonged drug-free breaks.

Metronomic chemotherapy has been defined as a variation of dose-dense therapy with the important exception that it is not necessarily dose intense, the cumulative dose might actually be significantly less or equal to MTD-based chemotherapy, thus reducing or perhaps even eliminating in some cases serious drug-induced toxicities, and hence the need for growth-factor support [6]. Unlike MTD chemotherapy that presumably mainly targets (proliferating) tumour cells, frequent or continuous low-dose chemotherapy appears to inhibit preferentially the endothelial cell activity of the tumours' growing vasculature [7]. The basis of this surprising selectivity may have a number of mechanisms.

One is believed to involve inhibition of tumour angiogenesis as a result of chemotherapeutic drug targeting of dividing endothelial cells found in growing tumour-associated blood vessel capillaries.

Moreover, human vascular endothelial cells in vitro are sensitive to the growth inhibiting effects of ultra low concentrations of paclitaxel, in contrast to many other normal cell types or tumour cell types. These effects can be amplified by long-term, continuous exposure, which can also result in apoptosis of endothelial cells [8]. Such effects may be secondary to induction of an endogenous inhibitor of angiogenesis, thrombospondin-1, induced by low-dose chemotherapy by as yet unknown mechanisms, rather than direct inhibition of endothelial cell growth, or survival [9]. In addition, the mobilization, viability and levels of angiogenesis contributing to circulating endothelial progenitor cells may be strongly suppressed, and in a sustained manner, by metronomic chemotherapy [10].

Among the numerous preclinical schedules using different cytotoxic drugs, the oral and daily low-dose administration of cyclophosphamide has been extremely successful in human prostate and breast cancer xenograft mouse models. It has also been successful in spontaneously arising islet cell pancreatic carcinoma, even when the treatment is initiated on advanced, late stage bulky tumours, at least when combined with a targeted anti-angiogenic drug and upfront MTD chemotherapy [11,12]. This efficacy is accompanied by absent or low-grade toxicity on tissues otherwise highly sensitive to the respective regimen of the same drug.

For these reasons we used oral daily low-dose cyclophosphamide for our patient, who because of her bad performance status was against undergoing another traditional line of chemotherapy. To our knowledge this is the first report showing the feasibility and the efficacy of cyclophosphamide metronomic chemotherapy for palliative treatment in advanced epithelial ovarian cancer. This is a simple therapy that can be administered on an outpatient basis. Our patient received treatment while performing normal daily activities at home and a high quality of life was maintained.

Probably this good clinical response was due to the anti-angiogenetic effect of cyclophosphamide in blocking progression of the disease without any toxicity, together with successful colostomy to improve the general condition of the patient.

The hypothetical anti-angiogenic and anti-tumour effects of low-dose metronomic chemotherapy regimens in mice can be amplified significantly by the concurrent administration of a second drug that is highly specific for activated endothelial cells (e.g., antibodies to vascular endothelial growth factor receptor-2 [VEGFR-2], PEX [C-terminal hemopexin-like domain of matrix metalloproteinase (MMP)-2], anti-VEGF antibodies, and anti-endoglin antibodies). Experiments testing such 'metronomic' schedules of chemotherapy alone or combined with anti-VEGF compounds showed promising anti-tumour activity [13]. A recent phase II study evaluated bevacizumab and low dose 'metronomic' oral cyclophosphamide and found encouraging response rates in 29 patients (6 partial response, 17 stable disease, 6 progressive disease) and progression-free survival at 6 months for 47% of the patients [14]. Despite the encouraging preliminary results reported at the 2005 Meeting of the American Society of Clinical Oncology, this non randomized study presents several limitations: the small number of patients evaluated, the lack of randomization that does not allow discrimination between the activity of 'metronomic' chemotherapy and that of the selective anti-VEGF compound; and, finally, the fact that no correlation with potential markers of angiogenic activity was determined. Currently no definitive clinical data are available because no randomized trial comparing conventional versus metronomic with dose-dense schedules has been concluded [15].

## Conclusion

In conclusion, low dose, oral CTX, demonstrated significant efficacy in this patient with pre-treated advanced ovarian cancer. Theoretically, treatments aimed at inhibiting angiogenesis should be chronically administered for a prolonged period. We think that use of oral CTX should be investigated further, with or without a second drug that is highly specific for activated endothelial cells, as a strategy against tumour progression after standard chemotherapy in patients who are platinum resistant with poor performance status.

## Competing interests

The author(s) declare that they have no competing interests.

## Authors' contributions

SR performed chemotherapy and decide to submit the manuscript for publication.

CG prepared the manuscript, reviewed the literature, prepared the figure and edited the report.

VE and SC performed surgical treatment.

All authors read and approved the final manuscript.

## Pre-publication history

The pre-publication history for this paper can be accessed here:
